# Applying symptom appraisal models to understand sociodemographic differences in responses to possible cancer symptoms: a research agenda

**DOI:** 10.1038/bjc.2015.39

**Published:** 2015-03-03

**Authors:** K L Whitaker, S E Scott, J Wardle

**Affiliations:** 1 Faculty of Health and Medical Sciences, School of Health Sciences, University of Surrey, Guildford, Surrey GU2 7XH, UK; 2 Unit of Social and Behavioural Sciences, King's College London Dental Institute, London SE5 9RW, UK; 3 Department of Epidemiology and Public Health, Health Behaviour Research Centre, University College London, London WC1E 6BT, UK

**Keywords:** cancer, symptom, bodily change, appraisal, patient presentation, appraisal interval, sociodemographics, inequalities

## Abstract

**Background:**

Sociodemographic inequalities in the stage of diagnosis and cancer survival may be partly due to differences in the appraisal interval (time from noticing a bodily change to perceiving a reason to discuss symptoms with a health-care professional). A number of symptom appraisal models have been developed describing the psychological factors that underlie how people make sense of symptoms, although none explicitly focus on sociodemographic characteristics.

**Methods:**

We therefore conducted a conceptual review synthesising all symptom appraisal models, and focus on potential links with sociodemographics that could be the focus of future research.

**Results:**

Common psychological elements across nine symptom appraisal models included knowledge, attention, expectation and identity, all of which could be sensitive to sociodemographic factors. For example, lower socioeconomic status (SES), male sex and older age are associated with lower health literacy generally and lower cancer symptom knowledge. Limited attentional resources, lower expectations about health and lack of social support also hamper symptom interpretation, and would be likely to be more prevalent in those from lower SES backgrounds. Symptom heuristics (‘rules of thumb') may lead to symptoms being normalised because they are common within the social network, potentially disadvantaging older populations.

**Conclusions:**

A better understanding of the processes through which people interpret their symptoms, and the way these processes differ by sociodemographic factors, could help guide the development of interventions with the aim of reducing inequalities in cancer outcomes.

In England there are notable socioeconomic inequalities in cancer survival (Rachet *et al*, 2010; Ellis *et al*, 2012), and cancer survival generally decreases with increasing age (Office for National Statistics, 2013). Lower socioeconomic status (SES), male sex and older age were risk factors for later stage at diagnosis across seven common cancers, and it was estimated that eliminating sociodemographic inequalities in England would result in 5600 fewer patients a year being diagnosed at an advanced stage, which would in turn translate into substantial improvements in cancer survival rates (Lyratzopoulos *et al*, 2013).

The majority of cancers are diagnosed following symptomatic presentation in primary care (Elliss-Brookes *et al*, 2012). The timing of help-seeking for potential cancer symptoms is therefore a potentially modifiable route to improving early diagnosis (Richards, 2009). The Model of Pathways to Treatment focusses on conceptualising and identifying potential areas of delay (Scott *et al*, 2012). This is a descriptive framework of events (e.g., detecting a bodily change), processes (e.g., patient appraisal) and intervals. The patient interval is described as including both an appraisal component (time from noticing a bodily change to perceiving a reason to discuss it with a health-care professional) and a help-seeking component (time from perceiving a reason to discuss symptoms with a health-care professional to first consultation). Variation in the patient interval has been highlighted as a potential source of inequalities in the stage of diagnosis for ‘easy-to-suspect' cancers (e.g., breast), because for patients subsequently diagnosed with these cancers the diagnostic interval tends to be short and equitable (e.g., Lyratzopoulos and Abel, 2013).

There is also evidence demonstrating that lower SES, male sex and older age are associated with delayed presentation for cancer symptoms (Ramirez *et al*, 1999; Macleod *et al*, 2009; Forbes *et al*, 2014). Data from the English Routes-to-Diagnosis project reported that lower SES and older age were also associated with a higher incidence of emergency diagnoses across cancer types (McPhail *et al*, 2013), also indicating that there may be differences in the way symptoms are appraised or acted upon.

Research into sociodemographic inequalities has tended to focus on health-care use post presentation (Smith *et al*, 2005; Macleod *et al*, 2009). However, the appraisal interval (before GP visit) has been estimated to account for over two-thirds of the patient interval (Ristvedt and Trinkaus, 2005). It is therefore important to try to understand how sociodemographic factors affect the processes of symptom interpretation, and the determinants of decisions to seek help. This could help in developing targeted interventions designed to reduce sociodemographic inequalities. This is particularly important at a time when reducing the proportion of cancer patients diagnosed as an emergency is a priority for contemporary health-care systems (Lyratzopoulos *et al*, 2014).

## Materials and methods

This literature review synthesises models of symptom appraisal and identifies elements of the models that may have application to sociodemographic differences. It is not a systematic review but rather a synthesis of models that highlight elements relevant to how people perceive, and act on, their symptoms. A number of different models of symptom appraisal may be relevant to unpacking patient response processes. In these models, symptoms (which may also be termed bodily sensations or changes) are considered ‘an outcome of a perceptual process' (Cioffi, 1991), and interpretation of bodily sensations is subject to complex psychosocial influences (Pennebaker, 1982; Cacioppo *et al*, 1986; Cioffi, 1991).

We used a keyword search of electronic databases (PubMed, Web of Science and PsychINFO) to identify relevant models using the terms symptom, somatic, illness, perception, appraisal, interpret and model. Additional models were found from reference lists. Inclusion criteria for models were broad and included the following: (1) the model has been published in English journals or books (no date limits were set); (2) the model started from before the presence of noticeable symptoms; and (3) the model was predominantly about how people notice and interpret symptoms. We did not include the Model of Pathway to Treatment because it is not a unique model of symptom appraisal in its own right but rather a framework in which existing psychological theories of appraisal and help-seeking are integrated. We do, however, include those symptom appraisal models that Scott *et al* (2012)integrated into the appraisal interval, and draw on the authors' overall discussion of symptom interpretation.

The aim was to provide a conceptual framework to help understand sociodemographic differences in symptom appraisal. We focussed principally on SES because lower SES is a risk factor for advanced stage at diagnosis across several cancer types (Lyratzopoulos *et al*, 2013), but we adopted a multi-faceted view incorporating occupational status, income, material resources, education and neighbourhood characteristics as markers of SES. We also explored evidence for age and sex differences, although the magnitude and direction of age and sex inequalities in the stage of cancer diagnosis varies by cancer type (Lyratzopoulos *et al*, 2013). The primary outcome was to identify potential implications of the concepts/models for understanding inequalities in the earlier diagnosis of cancer.

## Results

### Symptom appraisal models

The symptom appraisal models included in this review are summarised in Table 1. There are broad commonalities in their description of the process of symptom appraisal, which are categorised as detection of bodily changes, interpretation of bodily changes and responses to interpretation. These are briefly outlined below.

#### Detection of bodily changes

Detection of bodily changes is the starting point across models of symptom appraisal, variously referred to as ‘recognising a disturbance in the human system' (Teel *et al*, 1997), ‘internal somatic information' (Kolk *et al*, 2003), ‘somatic label' (Cioffi, 1991) and ‘situational stimuli' (Leventhal *et al*, 1997). Bodily changes may be general (e.g., fatigue) or localised (e.g., rash and lump). They could be visible (e.g., mole), palpable (e.g., lump) or audible (e.g., joint clicks). They may represent a difference in frequency or intensity of normal bodily sensations or a novel event (Scott *et al*, 2012). The common theme across symptom appraisal models is that for a bodily change to be detected it must be of sufficient magnitude or significance. External conditions may affect this; for example, in the Competition of Cues Theory, Pennebaker (1982) suggested that awareness of internal states is a function of the ratio of the quantity and salience of internal to external information. New bodily changes may go unnoticed if the external environment is high on stimulation, whereas fluctuations in normal bodily sensations may be noticed and attended to if the external environment is lacking (Cacioppo *et al*, 1986).

#### Interpretation of bodily changes

Following detection of a bodily change, individuals are assumed to go through a process of interpretation, which may involve labelling, categorising and evaluating the bodily change. Detection and interpretation will likely overlap, because bodily changes may become salient through interpretation (Alonzo, 1979; Pennebaker, 1982). For example, someone who considers they may have the flu may infer that they are feeling unusually hot. Interpretation is open to inaccuracy and is influenced by a range of factors such as knowledge, emotions (e.g., fear), co-morbidity, social context and previous experience. For example, a previous ‘false alarm' may influence the appraisal of new or recurrent symptoms, making a benign attribution more likely (Renzi *et al*, 2015).

#### Responses to interpretation

The final strand common to symptom appraisal models is the response to interpretation, defined as the actions and behaviours that follow the interpretation process. In terms of the appraisal interval, the end point is the decision that the symptom warrants medical attention (or not). If the interpretation process has resulted in a benign hypothesis (e.g., a change in the appearance of a mole interpreted as an insect bite), this will prolong the appraisal interval for persistent symptoms (Andersen *et al*, 1995; Walter *et al*, 2014). During the appraisal interval, other responses can also occur, including self-monitoring, self-management and seeking advice from family and friends (Scott *et al*, 2012). Behaviour resulting from symptom interpretation can also be avoidant rather than help-seeking, such as avoiding touching or looking at a symptom (Dingwall, 2001), or finding a distractor (Cioffi, 1991). Both the common sense model and the illness action model propose that output processes be subsequently appraised to see whether they have been successful (Leventhal *et al*, 1997; Dingwall, 2001). This self-regulation approach allows for ongoing cycles of symptom appraisal and re-appraisal.

### Elements of symptom appraisal models and their potential links with sociodemographic factors

**Figure 1:**
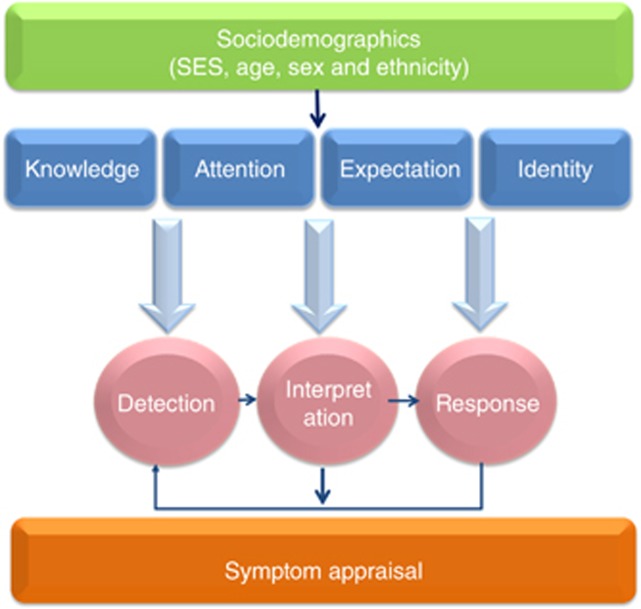
**The influence of sociodemographic factors and psychosocial ‘elements' on symptom appraisal.** Demonstration of how sociodemographic factors may influence the psychosocial ‘elements' of symptom appraisal. Responses to interpretation may be re-evaluated, leading to new cycles of detection and interpretation. Knowledge is defined as familiarity, awareness or understanding of bodily sensations acquired through experience or education. Attention is defined as focussing on relevant stimuli while ignoring distractors. Expectation encompasses pre-existing beliefs, contextual biases and general heuristics (i.e., shortcuts). Identity refers to the distinct characteristics of an individual and their role in society.

All of the symptom appraisal models identify factors that broadly resemble ‘knowledge', ‘attention', ‘expectation' and ‘identity' as determinants of progress along the detection–interpretation–response pathway (see Figure 1). Examples of these elements in relation to each model are summarised in Table 1, and are explained in detail below. Each section also explores how these factors could contribute to sociodemographic differences in symptom appraisal and provides some examples of possible future interventions (see Table 2). It is important to note that the different elements do not operate in silos: rather, they influence one another during the appraisal process. For example, health knowledge (e.g., that persistent cough can be an early warning sign of cancer) may be influenced by previous experience/expectations (e.g., I've previously had a cough like this and it turned out to be related to my asthma).

#### Knowledge

**Overview.** Across models the concept of ‘knowledge' is described as ‘exemplars', 'schemes', ‘schemas', ‘mental representations', ‘prototypes' and ‘awareness', but in all cases is conceived of a cognitive factor that guides the interpretation process. When bodily changes are detected, people use their existing knowledge to aid interpretation.

**Sociodemographics differences.** Population-based surveys in the United Kingdom using the cancer awareness measure (CAM; Stubbings *et al*, 2009) show that knowledge about cancer ‘warning signs' is lower in lower SES groups, men and older people (Wardle *et al*, 2001; Robb *et al*, 2009; Hvidberg *et al*, 2014). Modules of the CAM for specific cancer sites also show that knowledge of warning signs for lung, colorectal and cervical cancer is lower in lower SES groups (Power *et al*, 2011; Low *et al*, 2012; Simon *et al*, 2012). Men were also less likely to know the warning signs for colorectal cancer (Power *et al*, 2011). If sociodemographic characteristics are associated with being less likely to hold schemata for relevant ‘warning signs', this would influence the symptom interpretation process and in turn make help-seeking less likely. Consistent with this, lower awareness of cancer ‘alarm' symptoms is associated with a longer ‘anticipated' help-seeking interval (Robb *et al*, 2009; Quaife *et al*, 2014).

Older age, lower education, lower income and being male are also associated with lower health literacy (von Wagner *et al*, 2007), defined as ‘the capacity to obtain, process and understand basic medical information and services needed to make appropriate health decisions' (American Institute of Medicine, 2004). Lower health literacy may make it more difficult to understand and thus benefit from public health messages aimed at increasing knowledge, because concepts such as ‘early detection' are not well understood (Doak *et al*, 1996). However, whether health literacy is the primary explanation of sociodemographic differences in awareness of warning signs has not been investigated.

These results indicate that one avenue by which to reduce inequalities in earlier diagnosis is the targeting of particular demographic groups in which awareness and health literacy are known to be lower. In the United Kingdom, there are some campaigns targeting specific groups (e.g., breast cancer awareness campaign for women over 70 years; Cancer Research UK, 2014). If successful, these could be followed by campaigns targeting other symptoms or groups.

#### Attention

**Overview.** The detection phase of symptom appraisal is inherently linked to attention, because self-focussed attention is required to detect a symptom in the first instance. Attention is also important in interpretation because symptom appraisal is dependent on available cognitive resources to understand the explanation for the symptom and its implications. Kolk *et al* (2003) proposed that attention is influenced by the availability of internal resources for selective attention (i.e., focussing on relevant stimuli while ignoring distractors), the presence of external information (the events occurring in the person's environment) and negative affectivity. The Competition of Cues Theory also highlighted the effect of competing demands or external stressors in making it less likely that symptoms are noticed and interpreted (Pennebaker, 1982).

**Sociodemographics differences.** Several aspects of the ‘attention' element could relate in different ways to sociodemographics. Traits relevant to depleted attentional resources, such as ‘reactive responding' (an information-processing style characterised by reduced capacity for dealing with novel stressors) (von Wagner *et al*, 2011), have been linked with lower SES. Low consideration of future consequences has also been associated with lower SES (Guthrie *et al*, 2009). This may be because people from more deprived backgrounds need to concentrate on the present (von Wagner *et al*, 2011), or because there are so many external uncontrollable threats that internal attention appears unimportant (Nettle, 2010).

In line with the Cue Competition Theory, the theme of ‘competing demands' has been associated with lower SES, because demanding social environments often characterise lower SES households (Gallo, 2009). Competing family and work demands have also been raised by women as a reason for not seeking help for breast cancer symptoms (Facione and Facione, 2006), particularly among women aged 35–54 years (Grunfeld *et al*, 2003).

Women are shown to report more symptoms and visit the doctor more often than men (Vanwijk and Kolk, 1997). Pennebaker (1982) argued that there is not a simple correspondence between physiological change and symptoms; rather, women may be more attentive to their internal states. Evidence from the cancer literature has indicated that women were more likely to engage in actions to detect possible cancer symptoms (e.g., examining skin for changes) compared with men (de Nooijer *et al*, 2002; van Osch *et al*, 2007). Future work could explore other sociodemographic differences in body vigilance (Schmidt *et al*, 1997).

Negative effect may also contribute to SES differences in attention. According to the reserve capacity model, lower SES individuals experience more depression, anxiety and hostility compared with high SES individuals (Gallo, 2009). Negative effect makes symptoms more likely to be noticed, but more likely to be attributed to psychological causes (e.g., stress) (Kolk *et al*, 2003).

Targeting of attentional components may require a multi-faceted approach. For example, people low in consideration of future consequences could benefit from emphasis on the short-term benefits of presenting early with symptoms (e.g., reassurance) alongside the potential long-term gains (earlier diagnosis). If evidence supports the notion that there are individual differences in body vigilance, campaigns could be targeted at groups who are less likely to notice changes in their body, offering practical action plans to encourage them to make time to attend to their body.

#### Expectation

**Overview.** Expectation-based processes feature in a number of symptom perception models and refer to the contribution of beliefs and emotions in guiding the perception and interpretation of bodily changes (Petersen *et al*, 2011). Expectations can refer to pre-existing beliefs about bodily sensations, contextual biases or general heuristics (rules of thumb) that simplify symptom interpretation (Petersen *et al*, 2011). Expectations such as how long a symptom normally lasts (duration rule), how common symptoms are (prevalence rule) and how new or different they are (novelty rule) can influence appraisal, as can heuristics based on age (as individuals grow older they expect bodily changes to occur because of ageing rather than illness) and gender (e.g., women experiencing cardiac symptoms do not consider cardiac causes, as heart problems are expected to occur in men, and not in women) (Scott *et al*, 2012).

**Sociodemographics differences.** Sociodemographic characteristics have clear implications for both the prevalence rule and age or sex stereotypes. The prevalence rule highlights that, if symptoms are considered prevalent, people are less likely to appraise them as serious or as warranting medical attention (Scott *et al*, 2012). As well as attributing symptoms to age rather than illness, older people may experience more symptoms simultaneously due to co-morbidities, making symptom interpretation more difficult. The possibility of co-morbidity leading to misinterpretation of symptoms may also be more likely in lower SES groups, because they tend to have higher levels of symptoms (Pennebaker, 1982; Kroenke and Spitzer, 1998; Kolk *et al*, 2003). With regard to the influence of stereotypes, women are more likely to have advanced stage at diagnosis compared with men for bladder cancer (Barbiere *et al*, 2011), and one possibility is that they are more likely to misattribute their symptoms to common female conditions such as cystitis.

Expectation-based biases may require correcting with ‘myth buster' campaigns that highlight the pitfalls of making assumptions based on age or sex. Raising awareness of age-related risk of cancer may also militate against ‘normalising' attributions in the older population.

#### Identity

**Overview.** In the Symptom and Illness Attitude Model, Petersen *et al* (2011) argued that taking into account the complex nature of the self can help illuminate the process of symptom appraisal. They proposed that people have multiple identities that influence how they perceive bodily changes. For example, if ‘being a parent' is the most accessible identity, it may make it less likely that illness-related schemata are activated in the presence of bodily sensations. Other symptom appraisal models also emphasise the importance of identity. The common sense model of illness described the influence of ‘groups' and ‘roles' (e.g., negotiating the ‘sick role' alongside commitments associated with the ‘family role'). Alonzo's situational-adaption model emphasised that symptoms are always appraised within socially defined situations, and one key goal may be to ‘contain' symptoms and prevent them from having an impact on daily life by adopting processes such as normalising (Alonzo, 1979).

On the other hand, social situations may facilitate symptom interpretation and earlier diagnosis. In the illness action model, other people are considered to have a role in recognising and interpreting bodily changes (Dingwall, 2001). Wyke *et al* (2013) suggested that the influence of social structure can be direct (e.g., ability to obtain child care) or indirect (e.g., social networks' access to resources such as knowledge and health literacy). Qualitative research showed that discussing bodily sensations with others stimulated early detection (de Nooijer *et al*, 2001), and a review of help-seeking for lung symptoms concluded that social networks influenced the sanctioning of symptom seriousness in individuals who may otherwise be reluctant to perceive a reason to contact a doctor (Chatwin and Sanders, 2013). The potential impact of social facilitation on earlier diagnosis was highlighted by data from the United States, which showed that married patients were less likely to present with metastatic disease and less likely to die as a result of their cancer (Aizer *et al*, 2013).

**Sociodemographics differences.** Self-identity (i.e., the way people perceive themselves) is influenced by characteristics, including their sociodemographic profile. Self-identity is constructed within a social and cultural reality (Alonzo, 1979) and may also help explain cultural differences in symptom appraisal (Alonzo, 1979; Andersen *et al*, 2010).

Men and people from lower SES backgrounds rate their expected life expectancy as significantly lower compared with women and people from higher SES backgrounds (Wardle and Steptoe, 2003). Holding negative expectations about future health may affect symptom appraisal by making people more likely to ‘contain' or side-line worrying bodily changes by failing to acknowledge them as symptoms (Andersen *et al*, 2010). In support of this, UK data showed that people from lower SES backgrounds were less likely to consult compared with people from higher SES backgrounds when they appraised their symptoms as severe, but more likely to consult when they appraised their symptoms as trivial (Elliott *et al*, 2012).

The experience of others who have worse conditions/illnesses (more likely in lower SES environments and older populations) may also influence interpretation (i.e., the symptom does not seem so important anymore) (Petersen *et al*, 2011). Lower SES is also associated with lower social support, both emotional (e.g., providing encouragement) and instrumental (e.g., help with child care) (Taylor and Seeman, 1999), which may influence how people notice and interpret symptoms.

One possibility is that negative expectations of future health (based on self-identity) may contribute to inaction when people are faced with potential cancer symptoms. Modifying identity, particularly based on a sociodemographic group, is likely to be difficult. However, one possibility would be to increase the public's confidence in cancer as a curable and survivable disease, resulting in higher future expectations. Another possibility would be to harness the positive influence of social support in aiding symptom interpretation. Both of these suggestions may be well served at the community level, with involvement of potential users of the intervention from the start.

## Discussion

A synthesis of nine models of symptom appraisal identified common processes, which included knowledge, attention, expectation and identity. In some cases there is evidence of sociodemographic variation in these elements, which could explain the sociodemographic differences in responses to possible cancer symptoms. Lower symptom awareness and health literacy in men, older people and people from lower SES backgrounds may contribute to misinterpretation of possible cancer symptoms. Attentional aspects may have an impact because higher external demands and negative effects, particularly in those from lower SES backgrounds, make interpreting bodily changes challenging. Expectation-based biases resulting from co-morbidity, stereotypes and the age–illness rule influence symptom interpretation, and sociodemographic differences in identity and life experience may hamper the detection or appropriate interpretation of symptoms. Conversely, social sanctioning may facilitate recognition of symptom seriousness and encourage help-seeking in higher SES groups.

### Future research and interventions

The next step is to design studies within specific cancer types to test the mediation of sociodemographic differences by symptom appraisal processes. This cancer-specific approach will also allow for differential effects of sociodemographics to be observed according to cancer type. The resulting evidence base can be used to inform the development of targeted interventions, designed, for example, to make it more likely that people appraise their symptoms accurately (knowledge), are more vigilant for bodily changes (attention), are less pessimistic (expectation) and have a better understanding of their personal risk (identity), with the goal of reducing time to help-seeking.

### Limitations

This review provides a synthesis of symptom appraisal models to draw out common elements that may help understand inequalities in potential cancer symptoms. The primary aim was to highlight links between the psychological constructs from the models and demographic characteristics that could be the focus of future research. As the focus was on stimulating a research agenda, this review was not amenable to other types of review such as meta-analysis or meta-synthesis. In addition, because the literature review was not exhaustive, we may have omitted studies that would have provided a valuable contribution. The conceptual framework was developed with cancer in mind, but, as with models of help-seeking behaviour, may also be applicable to other illnesses (Scott *et al*, 2012).

We focussed more on SES than on age or sex because there is more consistent evidence that lower SES is associated with advanced-stage cancer (Lyratzopoulos *et al*, 2013). However, we also presented possible linkages with age and sex inequalities, although the direction of the effect is often dependent on cancer type (Lyratzopoulos *et al*, 2013). We did not discuss the influence of ethnicity due to scant evidence in the United Kingdom regarding the association between ethnicity, help-seeking and stage at diagnosis. It has also been suggested that ethnicity data are not well recorded (Jack *et al*, 2009, 2010), and, to date, the majority of help-seeking studies have been in ethnically homogeneous samples (Marlow *et al*, 2014). One strength of the proposed framework is that ethnicity can be readily incorporated at a later stage. The evidence presented in the review is mainly drawn from UK contexts, but as the elements extracted from symptom appraisal models are based on individual characteristics (e.g., knowledge) our framework should be generalisable to other health-care settings.

Despite the suggestion that the appraisal interval contributes significantly towards delay in symptomatic presentation, there is paucity of evidence distinguishing appraisal and help-seeking intervals. However, progress has been made. One qualitative study with ovarian cancer patients described how bodily sensations ‘transformed' into symptoms that were in need of further care (Brandner *et al*, 2014). It is important to remember that appraising symptoms as requiring medical attention does not necessarily equate to help-seeking; people may choose to avoid seeking help if they fear the outcome of medical consultation (Whitaker *et al*, 2015). Thus, our review must be considered in combination with the literature on models of help-seeking/consultation behaviour (Scott *et al*, 2012; Wyke *et al*, 2013) and wider integration with psychological theory (e.g., social cognitive theory; Bandura, 1997).

In a comparison between the common sense model of illness, the illness action model and the network episode model (the latter was excluded from the current review because the model starts after the presence of noticeable symptoms), Wyke *et al* (2013) highlighted similar elements to those discussed in this review, including the importance of knowledge and the role of social networks/candidacy in aiding symptom interpretation. Our discussion of the interaction of sociodemographic factors and elements such as ‘identity' in symptom appraisal provides a starting point for future research, but would benefit from further exploration. For example, ‘identity' as we discuss it overlaps with the concept of candidacy both in terms of people being ‘candidates' for certain diseases, including cancer (Macdonald *et al*, 2013), and in their candidacy for health care, which is determined by a complex interaction between themselves and cultural and organisational systems (Dixon-Woods *et al*, 2006; Mackenzie *et al*, 2013). The notion of candidacy may help explain the differential uptake of health care according to sociodemographic groups (Dixon-Woods *et al*, 2006).

## Conclusions

This conceptual review has synthesised models of symptom appraisal and highlighted elements that may help explain inequalities in the appraisal interval. It provides a framework to design studies that will provide a better understanding of the mechanisms through which people interpret their symptoms, and the relationship of these mechanisms to sociodemographic factors. This understanding should help guide the development of future intervention strategies to reduce inequalities in symptomatic presentation.

## Figures and Tables

**Table 1:** Summary of symptom appraisal models

**Symptom appraisal model**	**Authors**	**Central arguments/factors**	**Examples of elements with potential links to inequalities**
Situational adaption model	Alonzo, (1979)	Bodily sensations appraised within a social and cultural reality. Key assumption is that people are able to ‘contain' or side-line signs and symptoms within socially defined situations.	Identity: how bodily signs/sensations are understood as symptoms in the social and cultural arena.
Self-regulation theory/common sense model of illness	Leventhal *et al* (1997)	Representations of illness appraised and coped with according to self-regulation model. On detecting a bodily change, selection of coping response is driven by emotion and components of illness representations: identity (label), cause (attribution), timeline (duration), consequences (impact on daily life) and curability/control. Emphasises role of re-appraisals within a self-regulatory system.	Knowledge (i.e. symptom label), expectation-based biases, identity (e.g. roles).
Illness action model	Dingwall, (2001)	Sociological model of symptom perception. Bodily sensations may cause a ‘disturbance in equilibrium', which people seek to reconcile to continue as normal. Bodily sensations are perceived, interpreted and acted on and the outcome is evaluated.	Attention (competing demands), knowledge, identity (e.g. influence of others)
Cue competition theory	Pennebaker, (1982)	Appraisal of symptoms depends on cognitive resources available, when external sensory information is limited, more cognitive resources can be deployed to internal bodily sensations.	Attentional processes
Cognitive perceptual model of symptom perception	Cioffi, (1991)	Symptom appraisal comprised of meaning assignment, perceptual attention, and situational influences. Encompasses medical and psychosocial perspectives and role of attentional processing. Once bodily change detected, the search for a cause begins. Links to Pennebaker's theory.	Knowledge, attentional processes, expectation-based biases, identity
Psychophysiological comparison theory	Cacioppo *et al* (1986); Andersen *et al* (1995)	Assumes people are motivated to maintain a reasonable physiological condition. Proposes several principles of symptom appraisal including: attribution (cause), logical consistency (familiarity), optimistic bias, comorbidity (number of symptoms).	Knowledge, expectation-based biases (e.g. optimism/pessimism), identity
Symptom interpretation model (SIM)	Teel *et al* (1997)	Builds on Leventhal's Common Sense Model of Illness. Focuses on symptom experience from an intrapersonal perspective, where the interaction of knowledge and attribution of symptoms is considered critical.	Knowledge, expectation-based biases (i.e. heuristics, rules of thumb)
Kolk's symptom perception model	(Kolk *et al*, 2003)	Developed from Cioffi and Pennebaker's work. Highlights the effects of trait negative affect, selective attention and external stressors.	Attentional processes
Symptom and illness attitude model (SIAM)	Petersen *et al* (2011)	Synthesises/integrates models of illness and bodily sensations. Identifies moderators in forming mental representations of symptoms that may be promising targets for intervention.	Expectation-based biases (i.e. role of beliefs and emotion), identity (i.e. self-complexity).

**Table 2:** Examples of possible future intervention research

**Element**	**Recommendation**
Knowledge	Target public awareness campaigns at demographic groups where awareness and health literacy are known to be low
Attention	Emphasise short-term benefits of presenting with symptoms (e.g. reassurance), alongside long-term gains (e.g. early diagnosis)
	Target demographic groups where body vigilance is known to be lower, offering practical action plans
Expectation	‘Myth buster' campaigns targeted at age and sex stereotypes
	Raise awareness of age-related risk
Identity	Increase the public's confidence in cancer as a curable/survivable disease
	Harness positive influence of social support
	Community level interventions
